# phyloXML: XML for evolutionary biology and comparative genomics

**DOI:** 10.1186/1471-2105-10-356

**Published:** 2009-10-27

**Authors:** Mira V Han, Christian M Zmasek

**Affiliations:** 1 School of Informatics, Indiana University, Bloomington, IN 47408, USA; 2 Bioinformatics & Systems Biology, Burnham Institute for Medical Research, La Jolla, CA 92037, USA

## Abstract

**Background:**

Evolutionary trees are central to a wide range of biological studies. In many of these studies, tree nodes and branches need to be associated (or annotated) with various attributes. For example, in studies concerned with organismal relationships, tree nodes are associated with taxonomic names, whereas tree branches have lengths and oftentimes support values. Gene trees used in comparative genomics or phylogenomics are usually annotated with taxonomic information, genome-related data, such as gene names and functional annotations, as well as events such as gene duplications, speciations, or exon shufflings, combined with information related to the evolutionary tree itself. The data standards currently used for evolutionary trees have limited capacities to incorporate such annotations of different data types.

**Results:**

We developed a XML language, named phyloXML, for describing evolutionary trees, as well as various associated data items. PhyloXML provides elements for commonly used items, such as branch lengths, support values, taxonomic names, and gene names and identifiers. By using "property" elements, phyloXML can be adapted to novel and unforeseen use cases. We also developed various software tools for reading, writing, conversion, and visualization of phyloXML formatted data.

**Conclusion:**

PhyloXML is an XML language defined by a complete schema in XSD that allows storing and exchanging the structures of evolutionary trees as well as associated data. More information about phyloXML itself, the XSD schema, as well as tools implementing and supporting phyloXML, is available at .

## Background

Information that can be interpreted in a phylogenetic context is growing rapidly in both types and quantities, due to the advancement of large-scale studies such as metagenomics and phylogenomics [1,2]. Current formats for describing evolutionary trees are becoming increasingly inappropriate. The main limitation of present formats is the lack of standardized means to annotate tree nodes and branches with distinct attributes. In the case of species trees, these attributes are taxonomic names, branch lengths, and often (possibly multiple) support values (such as bootstrap values or posterior probabilities). Gene trees used in comparative genomics and phylogenomics applications additionally require fields for gene identifiers and potentially gene duplication events [3], whereas trees used in phylogeographic [4] applications require fields for geographic data. While some existing formats such as Nexus [5] or NHX (New Hampshire eXtended) [6,7] allow describing additional information associated with phylogenetic trees, these formats have been shown to be problematic in the extensibility or the interoperability as a standard. The complexity of the Nexus format has led to different parsers that only understand a subset of the format, and different programs that produce poorly formed outputs (although a XML based replacement for the Nexus format, named "NeXML", is being developed and is expected to alleviate problems stemming from the complexity of the Nexus format [8]). The NHX format, built as an *adhoc *extension to the Newick (New Hampshire) standard [9] has limits in the types of information it can incorporate, since it has been developed with one primary use case in mind - representing gene trees with inferred gene duplication events [3]. Previous proposals for a XML format for systematic data [10] never gained popularity, possibly due to a lack of supporting software.

Here we describe phyloXML, a new standardized format for phylogenetic documents that is based on the formal language of XML [11] and which is inspired by the XML tree representation described in [12] (this XML format is used as output format by the "Retree" program from the PHYLIP package [9]).

## Implementation

Along with the complete schema in XSD that defines the format of phyloXML, a number of tools have been implemented to support the reading and writing of phyloXML. The Java command-line tools "phyloxml_converter" can convert existing formats (Nexus, Newick/New Hampshire, and NHX) into phyloXML, and "decorator" helps the users insert various data types into a phyloXML tree. There are multiple tree-viewing programs that support the format, including Archaeopteryx [13] (the successor to the tree display tool ATV [7]) and TreeViewJ [14]. Furthermore, Archaeopteryx allows the user to easily convert phyloXML to Nexus, Newick/New Hampshire, and NHX and vice versa. So far, phyloXML support has been developed for three open source libraries for computational molecular biology and bioinformatics, namely BioPerl [15] (module Bio::TreeIO::phyloxml), BioRuby (module Bio::PhyloXML) [16], and Biopython (module Bio.Tree.PhyloXML) [17]. The XSD schema and links to supporting applications, together with more complex examples of phyloXML can be found at .

## Results and Discussion

PhyloXML is general, with over 20 different elements that encompass an extensive range of information (such as confidence values, sequence, and taxonomic data) that could be added to phylogenies. PhyloXML is extensible, containing legitimate grammar for user-defined contents, while it is also easy to expand the vocabulary of the schema without disrupting existing usage. Because the format is defined by a XML schema, phyloXML is also easy to validate and process. The structure of the document is readily parsed by any existing XML parser, while interpreting the content needs to be implemented depending on the use case. Because of the restrictive nature of the XML schema, unambiguous "well-formed" and "valid" documents will facilitate greater data exchange among users and programs that was not feasible before.

Similar to NHX, and unlike Nexus, the structure of phyloXML is phylogeny oriented rather than character oriented. The basic structure of a phyloXML document is a hierarchical cluster of recursive clades. Each clade corresponds to a node, and the set of clades that congregate at the root compose a phylogeny. Each clade element can also enclose nested elements that are annotations to the containing clade. This kind of hierarchical representation of the phylogeny and its corresponding annotations in each level is not only intuitive, but also naturally suitable for a description by XML. The following is an example of a phyloXML document describing a simple gene tree with three external nodes (for more examples, [see Additional file 1]).

<phylogeny rooted="true">

   <name>Alcohol dehydrogenases</name>

   <description>contains examples of commonly used elements</description>

   <clade>

      <events>

         <speciations>1</speciations>

      </events>

      <clade>

         <taxonomy>

            <id provider = "ncbi">6645</id>

            <scientific_name>Octopus vulgaris</scientific_name>

         </taxonomy>

         <sequence>

            <accession source="UniProtKB">P81431</accession>

            <name>Alcohol dehydrogenase class-3</name>

         </sequence>

      </clade>

      <clade>

         <confidence type="bootstrap">100</confidence>

         <events>

            <speciations>1</speciations>

         </events>

         <clade>

            <taxonomy>

               <id provider = "ncbi">1423</id>

               <scientific_name>Bacillus subtilis</scientific_name>

            </taxonomy>

            <sequence>

               <accession source="UniProtKB">P71017</accession>

               <name>Alcohol dehydrogenase</name>

            </sequence>

         </clade>

         <clade>

            <taxonomy>

               <id provider = "ncbi">562</id>

               <scientific_name>Escherichia coli</scientific_name>

            </taxonomy>

            <sequence>

               <accession source="UniProtKB">Q46856</accession>

               <name>Alcohol dehydrogenase</name>

            </sequence>

         </clade>

      </clade>

   </clade>

</phylogeny>

Application specific data types that are not covered by the schema are supported by phyloXML, explicitly as reserved <property> elements, as well as extensions that can be defined by the user. <property> provides an interface for custom typed and referenced data. The <property> elements can be applied to the <phylogeny> itself, the <clade>, or the parent branch inherent in each clade. The recursive structure has no bound for depth or breadth, and the same element can be attached to a node multiple times with different values as long as it complies with the schema. For example, phylogenies that are built as a consensus of multiple approaches can have multiple <confidence> elements attached to the same clade to describe the support values resulting from the different methods. Molecular sequence can be easily associated with a certain <clade> by the <sequence> element, and not only the raw sequence data but also complex annotations can be added to the sequence using <annotation>, <sequence_relation> (used to describe orthologous and paralogous relations, for example) and <domain architecture>. Table 1 describes some of the elements that are supported to annotate evolutionary trees.

**Table 1:** phyloXML elements and attributes summary

**Element/Attribute**	**Description and Sub-elements**
**phylogeny**	Represents a phylogeny, contains clades.
**clade**	Used recursively to represent node of a phylogeny.
**taxonomy**	Represents taxonomic information.
	**id**
	**code**
	**scientific_name**
	**common_name **
	**synonym**
	**authority**
**sequence**	A gene or protein associated with a clade.
	**symbol**
	**accession**
	**name**
	**location**
	**mol_seq**
	**uri**
	**annotation**
	**domain_architecture**
**events**	Events at a clade.
	**type**
	**duplications**
	**speciations**
	**losses**
	**confidence**
**annotation**	Annotation of sequence.
	**desc**
	**confidence**
	**property**
	**uri**
**property**	Typed and referenced mixed (free text) content.
**uri**	Uniform resource identifier (e.g. a URL).
**confidence**	Statistical confidence.
**distribution**	Geographic distribution of the items of a clade.
**date**	Date associated with a clade.
**sequence_relation**	Typed relationship between two sequences.
**clade_relation**	Typed relationship between two clades.
**id_ref**	Attribute, used together with id_source to describe relations between various elements.
**id_source**	Attribute, used together with id_ref to describe relations between various elements.

Representative elements and attributes of phyloXML (version 1.10) are shown. XML element/attribute names are in bold letters.

While the most straightforward structure of the document is the hierarchy of nested clades that automatically describes the topology of the phylogeny, it is also possible to describe the topology in a flat manner using the attributes id_ref and id_source. Id_source is an optional attribute that assigns a unique id to a <clade>, <taxonomy> or a <sequence>. Elements with an attribute of id_ref will be associated with the specific element that has the same value of id_source identifier. Using id_ref and id_source provides much flexibility in the structure of the document, and allows the representation of network topologies that cannot be represented with a hierarchical structure.

In the following, we compare and contrast key features of the phyloXML standard with those of the NeXML format currently being developed [8]. One significant difference between phyloXML and NeXML is that phyloXML provides predefined elements for data elements commonly used in phylogenetics, phylogenomics, and comparative genomics (such as elements for taxonomic and sequence information). In contrast, NeXML (in its most current version as of this writing) approaches this by providing meta elements which are intended to be compliant with RDFa recommendations so that they can be expanded to RDF triples by an XSL stylesheet [18,19]. This mechanism essentially allows expandable key/value attachments for various elements of an evolutionary tree, which are mediated by ontologies and which can be expanded to RDF. The obvious advantage of such an approach lies in its flexibility and in the fact that it allows representing unforeseen types of data and lends itself well to knowledge integration. On the other hand, different producers of NeXML formatted data might represent common data elements differently, in particular if they rely on different ontologies or if no commonly used ontology has yet been established for the problem domain, thus hampering the stated goal of interoperability for documents containing phylogenetic trees annotated with more than just basic OTUs (NeXML)/clade names (phyloXML). Due to the fact that phyloXML provides predefined elements, interoperability for documents containing commonly used types of data is guaranteed. Another advantage of explicitly modelling common elements in the XSD schema versus relying on key/value attachments mediated by ontologies is that this approach does not introduce dependencies on additional resources. To achieve the flexibility to represent data not modelled in the current XSD schema, phyloXML employs a two pronged strategy. Firstly, <property> elements can be used to store data not covered by the current schema. In fact, the "ref" attribute of <property> elements allows for mediation by ontologies, very similar to the approach used in NeXML. Secondly, XML is inherently extensible thus allowing the incorporation of data from other XML languages as well as extension of the phyloXML standard itself (in the form of future versions). Another difference between phyloXML and NeXML is in the structure of data representation. In NeXML, the data associated with the nodes are separated out of the tree into a tabulated structure; while in PhyloXML all the data associated with the nodes are within the tree structure itself. For this reason, the NeXML lends itself well to statistical approaches where the user treats the data as a list of samples. PhyloXML is convenient to use in algorithmic procedures where the data associated with the nodes are updated through multiple iterations of tree traversing, e.g. Expectation-Maximization across the tree. This difference is more of interest to the software developer who needs to think of how the data should be structured. Of course, both formats can ultimately be parsed and stored into any type of data structure, but one may be more suited for and easier to handle in certain approaches than the other. A third key difference between the two formats is that NeXML, like NEXUS, attempts to model all elements associated with phylogenetic inference, such as characters (molecular sequences, categorical data or continuous data), substitution models, and evolutionary trees. PhyloXML, in contrast, focuses on evolutionary trees with associated data only, thus simplifying the creation of compliant parsers and corresponding data structures.

Since phyloXML was not devised as input format for phylogeny inference software, we decided not to provide the means to store multiple sequence alignments as separate elements. In its current version (1.10, as of this writing), phyloXML only allows storing aligned molecular sequences via the <molecular_sequence> sub-element of <sequence> (with the "is_aligned" attribute set to "true"). Due to the extensible nature of XML, it is straightforward to add additional elements and sub-elements in future versions of phyloXML depending on user needs, without running into compatibility issues with existing software implementations.

The complete schema in XSD defining the phyloXML format, as well as software to visualize phyloXML formatted data is available at  under the open source LGPL license.

## Conclusion

We developed phyloXML, an XML language designed to describe phylogenetic trees and associated data. PhyloXML provides elements for commonly used features, such as taxonomic information, gene names and identifiers, branch lengths, support values, and gene duplication and speciation events. Using these standardized elements allows interoperability between various applications and databases. Furthermore, both due to extensible nature of XML itself and the provision of <property> elements by phyloXML, extensibility as well as domain specific applications are ensured.

We also developed a number of software applications to read, write, convert to and from, and visualize phyloXML formatted data. Furthermore, phyloXML is supported by the BioPerl [15], BioRuby [16], and Biopython [17] open source libraries. In practice, phyloXML has already proven valuable in research on regulatory network evolution [20,21]. In these studies evolutionary tree nodes were associated with such distinct data fields as taxonomic information, protein names, protein functions, domain-architectures, and gene duplications. PhyloXML provided a convenient and transparent means to store, visualize, and analyze these data in a phylogenetic context, leading to new biological insights.

## Availability and requirements

• **Project name: **phyloXML

• **Project home page: **

• **Operating system(s): **Platform independent

• **Programming language: **XML, Java, Perl (BioPerl), Python (Biopython), Ruby (BioRuby)

• **License: **GNU LGPL

• **Any restrictions to use by non-academics: **none

## List of abbreviations

**NHX**: New Hampshire eXtended; **OTU**: Operational Taxonomic Unit; **RDF**: Resource Description Framework; **RDFa**: Resource Description Framework in attributes; **XML**: Extensible Markup Language; **XSD**: XML Schema Definition; **XSL**: Extensible Stylesheet Language.

## Authors' contributions

MVH developed the phyloXML support in BioPerl and drafted the manuscript; CMZ developed the phyloXML format and its implementation in Java and helped to draft the manuscript. Both authors read and approved the final manuscript.

## Supplementary Material

Additional file 1 **phyloXML examples**. A file containing some simple evolutionary trees demonstrating select phyloXML features (the phylogenies can be displayed with Archaeopteryx, available at ; the XML itself can be viewed with any text or XML editor). Click here for file
